# Improving adolescent mental health and resilience through a resilience-based intervention in schools: study protocol for a randomised controlled trial

**DOI:** 10.1186/1745-6215-15-289

**Published:** 2014-07-18

**Authors:** Julia Dray, Jenny Bowman, Megan Freund, Elizabeth Campbell, Luke Wolfenden, Rebecca K Hodder, John Wiggers

**Affiliations:** 1 Hunter New England Population Health, Hunter New England Local Health District, Locked Bag 10, Wallsend, NSW 2287, Australia; 2 The University of Newcastle, Callaghan, NSW 2308, Australia; 3 Hunter Medical Research Institute, Locked Bag 1, Hunter Region Mc, NSW 2310, Australia

**Keywords:** adolescence, resilience, school, mental health, risk, intervention, prevention

## Abstract

**Background:**

Research investigating the effectiveness of universal interventions to reduce the risk of mental health problems remains limited. Schools are a promising setting within which adolescents can receive interventions aimed at promoting their mental health. The aim of this study is to assess the effectiveness of a resilience-based prevention-focused intervention in reducing the risk of mental health problems among adolescents attending secondary school in socio-economically disadvantaged areas.

**Methods/design:**

A cluster randomised control trial will be conducted, with schools as the unit of randomisation. Initially, 32 secondary schools will be randomly allocated to a control or intervention group (12 control and 20 intervention). An intervention focused on improving student internal and external resilience factors will be implemented in intervention schools. A survey of students in Grade 7 in both intervention and control schools will be conducted (baseline) and repeated three years later when the students are in Grade 10. The Strengths and Difficulties Questionnaire will be used to measure the risk of mental health problems. At follow-up, the risk of mental health problems will be compared between Grade 10 students in intervention and control schools to determine intervention effectiveness.

**Discussion:**

The study presents an opportunity to determine the effectiveness of a comprehensive resilience-based intervention in reducing the risk of mental health problems in adolescents attending secondary schools. The outcomes of the trial are of importance to youth, schools, mental health clinicians and policymakers.

**Trial registration:**

Australian New Zealand Clinical Trials Registry, ACTRN12611000606987, registered 14 June 2011.

## Background

Globally, the mental health of young people has been identified as a major area of health concern [1], with an estimated 10 to 20% of children and adolescents reported to have mental health problems [2]. In Australia, just over a quarter of young people aged 16 to 24 years report to have experienced a mental disorder in the past 12 months [3], with the prevalence of such disorders decreasing as age increases [3].

For adolescents, the risk factors for the development of mental health problems include social isolation, academic pressures, low self-esteem and poor body image [4,5], as well as health risk behaviours such as drug and alcohol use [6-8]. Research indicates mental health problems may also develop as a result of adversity, such as trauma or a stressful life event, and that childhood adversities can have significant and lasting negative effects on mental health that persist into adulthood [9-12].

Not all young people who experience disadvantage or adversity experience negative mental health outcomes. The concept of resilience provides one possible explanation for the ability of some individuals to maintain positive mental health in the face of adverse life circumstances [13]. Whilst often an inconsistently defined construct [14,15], the concept involves the ability, when faced with stress or adversity, to actively employ individual traits (internal factors) and wider social, community and environmental supports (external factors) to return to or maintain a positive state of mental health and functioning [16]. Internal resilience factors include personal strengths and factors such as self-efficacy and problem-solving skills [17-19]. External resilience factors include meaningful school, home and community participation, and prosocial peers [18,19].

Resilience in the context of adolescence and mental health is defined as a process by which risks are encountered, and assets or resources (internal and external resilience factors) are used to avoid a negative outcome, such as mental health problems [20-22]. Previous research in this area is limited; however, it suggests that high levels of resilience may prevent the development of mental health problems in adolescents [23]. In a study of 307 Norwegian adolescents aged 14 to 18 years, higher resilience scores were associated with lower scores for levels of depression, stress, anxiety and obsessive–compulsive symptoms [23]. Such an association was also found in relation to depressive symptoms in a separate sample of 387 Norwegian adolescents aged 13 to 15 years [24], supporting the suggestion that fostering resilience may prevent the development of mental health problems in adolescents [23].

Schools provide an opportune setting in which interventions to reduce the risk of mental health problems and to promote the resilience of adolescents may take place [25]. Positive outcomes with respect to both participant resilience factors and aspects of mental health have been reported in the limited number of school-based interventions that have to date adopted a resilience approach to target both outcomes. For example, the Penn Resiliency Program, a group cognitive-behavioural intervention delivered in selected schools, has been found to reduce depressive symptoms across early to mid-adolescence [26,27]. Similarly, the Asia-Pacific Resilience Project, a school-based resilience program, has been found to be effective in reducing mental health problems for younger children in Grades 1 to 6 [28]. In the authors’ knowledge, however, no such studies have utilised randomised controlled study designs, included a comprehensive measure of multiple external and internal resilience factors, and additionally assessed the risk of a range of mental health problems.

Given the identified gaps in research surrounding mental health and resilience in young people, a study is planned to assess the effectiveness of a comprehensive resilience-based prevention-focused intervention. The intervention is designed to improve student resilience factors and reduce the risk of mental health problems of adolescents attending secondary school in socio-economically disadvantaged areas. It is hypothesised that at follow-up, Grade 10 students in intervention schools will have a lower likelihood of being at risk of mental health problems compared to Grade 10 students in control schools.

## Methods/design

### Study design

**Figure 1:**
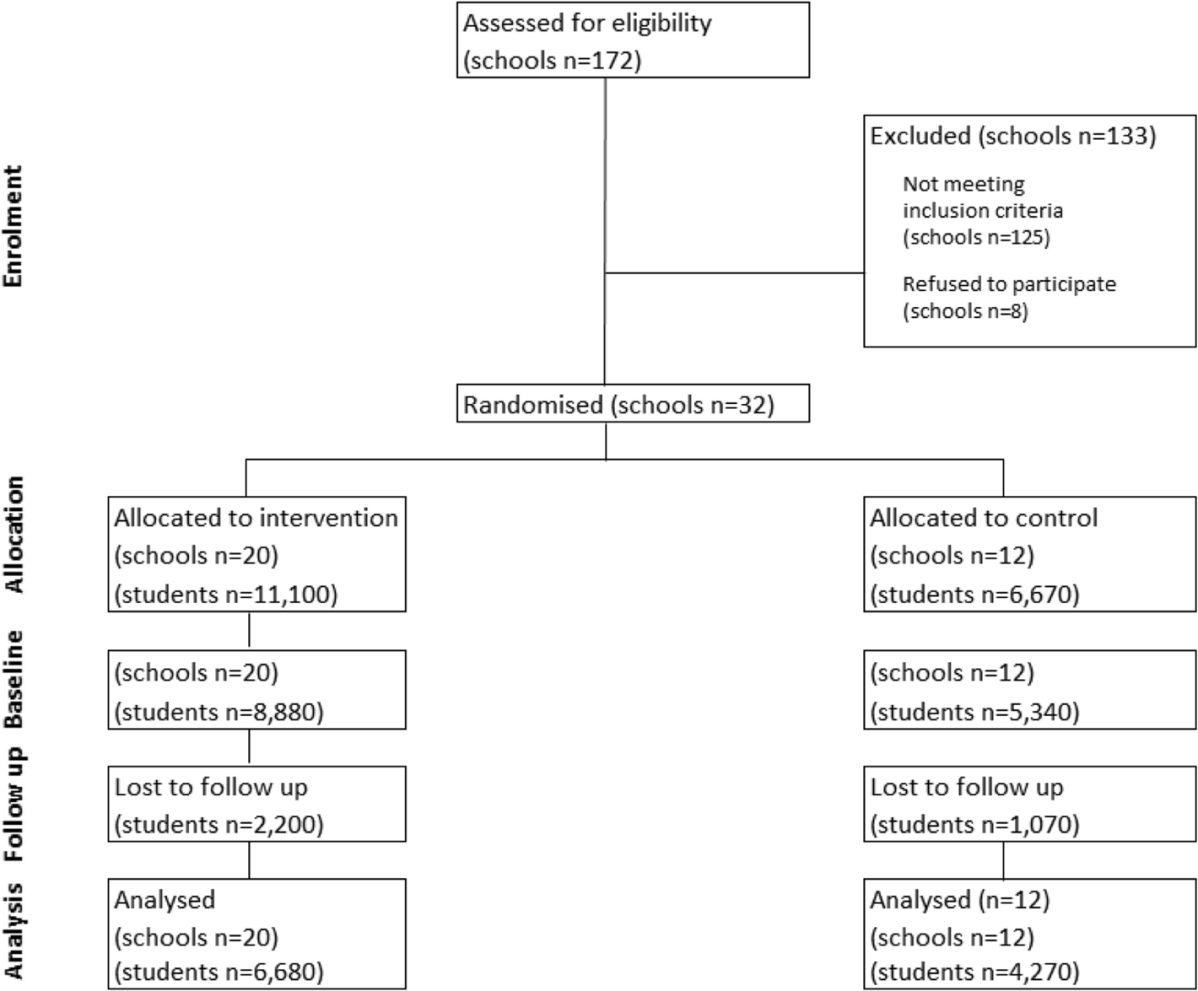
**Estimated CONSORT flow diagram for the schools’ progress through the trial phases (Hodder et al. 2012) **[29]**.**

A cluster randomised control trial design (Figure 1) will be conducted. The unit of randomisation will be the school. Initially, 32 schools in socio-economically disadvantaged areas will be randomly selected to participate in the study and randomly allocated to either the control (12 schools) or intervention group (20 schools). Web-based surveys will be conducted with all consenting Grade 7 students at baseline in 2011. Follow-up data will be collected from the same cohort of students three years later in 2014, when the students are in Grade 10.

The trial has been approved by the Hunter New England Health Human Research Ethics Committee (Ref no. 09/11/18/4.01), the University of Newcastle Human Research Ethics Committee (Ref no. H-2010-0029), the Aboriginal Health and Medical Research Council (Ref no. 776/11), the New South Wales Department of Education and Training State Education Research Approval Process (Ref no. 2008118), and the relevant Catholic Schools Offices. The trial is registered with the Australian New Zealand Clinical Trials Register (Ref no. ACTRN12611000606987).

### Participants

#### School sample

The study will be conducted within one Local Health District in New South Wales (NSW), Australia. The district covers an area of approximately 130,000 square km [30], has a large metropolitan centre, large regional areas and many smaller rural and remote communities [31].

The study will be conducted in secondary schools, both Catholic and government, within the study district. To serve as a sampling frame, a list of all schools in the study area will be obtained from the NSW Department of Education and Training and from relevant regional Catholic School Offices. Eligible schools will be located within a disadvantaged Local Government Area (using the Socio-Economic Indexes for Areas (SEIFA), which are indexes of relative socio-economic advantage and disadvantage by Local Government Area) [32], have a secondary student population of 400 students or more, have enrolments in Grades 7 to 10, and be co-educational. Central schools, boarding schools and schools that are entirely special needs or selective in nature will be ineligible.

#### School recruitment

Briefing meetings will be used to inform principals of eligible schools regarding the study, prior to the issuing of invitations to participate. Following this, eligibility interviews with school principals will be used to ascertain current strategies used to promote resilience within the schools. Schools identified as having implemented strategies across Grades 7 to 10 addressing each domain of the Health Promoting Schools framework (curriculum, teaching and learning; ethos and environment; and partnerships and services) [33] will be excluded.

Using a random number function (in Microsoft Excel), an independent statistician will be employed to order eligible schools. An invitation to participate will be sent to principals of the first 32 randomly selected secondary schools. Invitation letters will be emailed to inform principals of the study and request written consent for school participation. One week from the emailing of the information statements, principals who have not provided a response will be contacted by research staff to discuss any questions related to the study and to prompt a written response. At two weeks from the initial invitation, additional prompts will be made by research staff to principals still to reply. In the event that a school does not respond to the invitation or declines to participate, the school recruitment process described above will be repeated, with the next identified eligible school invited to participate. The process will be continued until 32 schools have been recruited.

#### Random allocation of schools

Following recruitment of the 32 schools, the sample will be stratified by school size (medium-sized schools have 400 to 800 students and large schools >800) and by engagement in a national government funding initiative directed at schools in disadvantaged areas [34]. Random allocation will be completed using Microsoft Excel to assign schools to the intervention or control group in a 20:12 block design ratio. Schools, parents of students and enrolled students will not be blinded to study group allocation.

#### Student sample

Students will be eligible to participate if enrolled in Grade 7 (first year of high school, typically aged between 12 and 13 years), and enrolled in a participating school. At baseline, it is estimated that approximately 3,600 Grade 7 students will be eligible to participate.

#### Student recruitment

Parental consent will be required for student participation in the evaluation component of the study. To maximise parental consent for child participation, a number of strategies will be utilised [35]. To maximise dissemination of study information, schools will be provided with information to share with the school community through existing school communication channels (i.e. newsletters, staff development days, staff and school bulletin boards, assemblies, community and parent gatherings). Study information packs will be mailed to parents. Packs will contain a cover letter formatted on the school letterhead from the principal, a detailed study statement for parents, a simplified study statement for students, a consent form requiring a parental signature for child participation in the study, and a reply paid envelope for return of the parental consent form. Within the parent study statement, parents will be provided information regarding a free call message service that they can call if they do not wish to have further contact from the research team.

Two weeks following the initial mailing of the study information packs, school-affiliated staff will telephone non-responding parents. During the call, parents will be asked to provide verbal consent or non-consent for their child to participate. For parents who provide verbal consent, replacement study information (a parent study statement, consent form and reply paid envelope) will be provided by mail. Additionally, informed student consent for participation in the evaluation component of the study, will be required from each participant prior to completion of student surveys at each point of data collection.

### Intervention

#### Intervention content

The multi-strategy resilience-based prevention-focused intervention will be implemented at a whole-school level (all students Grades 7 to 10). The intervention will incorporate a range of programs and strategies targeted at enhancing both the internal and external resilience factors of students in each of the three Health Promoting Schools domains (curriculum, teaching and learning; ethos and environment; and partnerships and services) [33]. A review of school-based programs by the World Health Organisation found that school-based interventions that adopted the Health Promoting Schools approach, and included intervention components in more than one school domain, to be most effective in achieving beneficial outcomes [36].

Intervention programs and strategies will be delivered and/or facilitated by the schools, rather than the researchers. Consequently, specific strategies and programs implemented may vary across schools. However, schools will be required to meet prescribed intervention standards when selecting and implementing resilience strategies. The intervention will involve the delivery of a range of unspecified evidence-based programs. The programs MindMatters [37,38] and Resourceful Adolescent Program [39] are listed in the next section (see ‘Health-promoting intervention strategies targeting resilience’ section) as examples of existing resilience programs suitable for selection by intervention schools. No specific evidence-based programs are mandatory for implementation within intervention schools.

#### Health-promoting intervention strategies targeting resilience [29]

Strategies relating to curriculum, teaching and learning:

• 100% of students in Grade 7 to 10 receive a minimum of 12 age-appropriate resilience lessons across subjects (e.g. implementation of MindMatters curriculum resources) [37,38].

• 100% of students in Grade 7 to 10 receive an additional 9 hours of non-curriculum-based resilience programs (e.g. implementation of the Resourceful Adolescent Program) [39].

Strategies relating to ethos and environment:

• Rewards and recognition program implemented across the whole school.

• Peer support or peer mentoring programs implemented across the whole school.

• Anti-bullying programs implemented across the whole school.

• Cultural awareness program implemented across the whole school.

• Teachers offered training to implement effective pedagogy within learning environments (e.g. MindMatters Teaching and Learning for Engagement) [37,38].

Strategies relating to partnerships and services:

• Promotion and engagement of local community organisations, groups and clubs in the school (e.g. charity organisations, and church and sporting groups).

• Promotion and engagement of health and community services in the school (e.g. Youth, and Child and Adolescent Mental Health Services).

• School implements strategies to increase parental involvement in the school (e.g. school events and effective parent communication strategies).

• School promotes strategies to address students’ resilience at home (e.g. newsletters regarding enhancing student resilience).

### Intervention adoption strategies

Previous critical evaluations of school-based health promotion strategies have identified adoption and implementation difficulties, and made recommendations for implementation strategies and intervention qualities deemed most effective for school-based interventions with a mental health focus [37,38,40,41]. A number of such intervention adoption strategies will be employed to facilitate intervention implementation and are listed below.

#### School intervention officers

School intervention officers will be employed at a ratio of one per four schools during the intervention period. The role of the intervention officers is to support schools in resilience intervention planning and data collection. School intervention officers will also be responsible for monitoring and maintenance of project records, and feedback of progress to schools, including specific feedback involving data obtained from student surveys. The intervention officers will not be involved in the direct delivery of programs and strategies to students.

#### Monitoring and feedback

Feedback on intervention progress will be delivered to school principals, other school staff, executive staff from the NSW Department of Education and Training, and relevant regional Catholic School Offices, on a regular basis.

#### Financial resources

Schools will be allocated AU$2,000 of funding annually, for the duration of the study, to support implementation of the resilience strategies. This funding can be expended on a range of purposes including teacher professional development; training in effective pedagogy for enhancing student resilience [42] and mental health literacy for both students and staff [43]; teacher relief to participate in training or intervention planning; and the purchase of evidence-based resilience programs or materials.

#### Cultural advice

An additional AU$2,000 will be allocated to schools annually to support Aboriginal student resilience. An Aboriginal Cultural Steering Group will be consulted for the duration of the research project. The purpose of the Steering Group will be to provide an opportunity for relevant Aboriginal cultural perspectives, advice, guidance and direction to influence the design, implementation, evaluation and dissemination of all project elements.

#### School core team

To encourage ownership and leadership in the implementation of the intervention within schools, a school core team will be established [37]. This may be formed through the enhancement of an existing leadership group within the school. Membership should include the allocated school intervention officer, student leaders, school staff including the school liaison officer and a minimum of one executive school staff member (e.g. principal, deputy principal and/or head of faculty).

#### Structured planning process

A structured planning process will be implemented to facilitate the development of a tailored intervention plan for each school. Prior to program implementation, a needs assessment will be conducted within each school to identify school-specific resilience needs, concerns and opportunities, and inform planning of strategies targeting student resilience. The assessment will consist of a student survey of both internal and external resilience factors for all students in Grades 7 to 10, as well as a survey of the school environment completed by executive school staff (e.g. principal, deputy principal and/or head of faculty) to identify existing school policies, practices and curriculum that may potentially impact student resilience (e.g. strategies in place that could enhance external resilience such as student empowerment programs or peer mentoring programs).

A detailed implementation guide will be provided to all schools outlining the intervention planning process along with a matrix of existing evidence-based resilience programs (e.g. MindMatters [37,38], SenseAbility [44], and Rock and Water [45,46]). Planning workshops will be held in each school with school staff and parents, and with other interested school community organisations and community members invited to attend and contribute to the sessions. Data collected during the needs assessment will be presented and discussed. From the workshops, each school will develop a tailored intervention plan to be endorsed by the school executive. Where possible, intervention plans and strategy implementation will be integrated into existing school governance, welfare and planning processes to ensure there is the minimum burden on schools during the implementation period.

### Control group

Control schools will continue to follow existing school policies and provide students with regular planned curriculum and non-curriculum activities. Baseline and follow-up student survey reports will be provided to all control schools following survey completion. Upon conclusion of the research project, all printed intervention resources will be provided to control schools.

### Data collection procedures

Students will complete an online survey during class time. Surveys will take place at both baseline and follow-up, under the supervision of research and school staff, and will take approximately 25 minutes.

For intervention schools, school characteristics (including major staff changes or adverse events) and implementation of intervention strategies will be monitored using the project records throughout the intervention period.

### Measures

#### Student demographics

The online student survey will contain demographic items including age, gender, grade, Aboriginal and/or Torres Strait Islander status, residential postcode, languages spoken at home and other cultural background.

#### Primary outcome: risk of mental health problems

Risk of mental health problems will be measured using the youth self-report version of the Strengths and Difficulties Questionnaire (SDQ) [47,48]. The SDQ consists of five subscales: emotional symptoms (five items), conduct problems (five items), hyperactivity/inattention (five items), peer relationship problems (five items) and prosocial behaviour (five items). Statements are rated on a three-point Likert scale: 0 (not true), 1 (somewhat true) and 2 (certainly true); with a small number of items negatively worded and reverse scored. Student scores from the 25 individual questions that are in each of the SDQ subscales will be used to calculate five subscale scores, with 0 to 10 being the possible range of scores for each subscale. High scores on the first four subscales listed indicate difficulties, with high scores in the final subscale (prosocial behaviour) reflecting strengths [49]. Four of the five subscale scores (emotional symptoms, conduct problems, hyperactivity/inattention and peer relationship problems) will be added to determine a total difficulties score (total SDQ) with a range of 0 to 40, with the total SDQ score being the primary trial outcome. The score for the fifth subscale (prosocial behaviour) is excluded from the calculation of the total difficulties score, as the presence or absence of prosocial behaviour is not clearly indicative of the presence or absence of psychological difficulties [47,48]. Reliability has been demonstrated for the youth self-report version of the SDQ in relation to use of the total SDQ score (Cronbach’s *α* = 0.80 to 0.82) [48,50], and the five SDQ subscales: emotional symptoms (*α* = 0.66 to 0.75), conduct problems (*α* = 0.60 to 0.72), hyperactivity/inattention (*α* = 0.67 to 0.69), peer relationship problems (*α* = 0.41 to 0.61) and prosocial behaviour (α = 0.65 to 0.68) [48,50]. Additionally, the youth self-report version of the SDQ has demonstrated validity when used for assessing the risk of mental health problems [48-50], and in the comparison of pre- and post-intervention scores, in adolescents [51].

#### Secondary outcome: resilience

Student internal and external resilience factors will be measured using the Resilience and Youth Development Module of the California Healthy Kids Survey (CHKS) [52,53]. The survey is one of the few to demonstrate conceptual adequacy by examining resilience using a multi-level approach [54]. Items within the survey measure six internal resilience factor subscales and eight external resilience factor subscales, which have demonstrated adequate reliability [19]. The internal resilience subscales include items addressing co-operation and communication (two items), self-efficacy (four items), empathy (three items), problem-solving (three items), self-awareness (three items) and goals and aspirations (three items). The external resilience subscales include items addressing school support (six items), school meaningful participation (three items), community support (six items), community meaningful participation (three items), home support (six items), home meaningful participation (three items), peer caring relationships (three items) and prosocial peers (three items). Students will be asked to respond to all items using a four-point Likert scale: 1 (never true), 2 (true some of the time), 3 (true most of the time) and 4 (true all of the time). Scores from individual survey items are averaged to calculate scores for each of the 14 resilience subscales. Scores from each of the internal resilience subscales and external resilience subscales are averaged to calculate a total internal resilience score and a total external resilience score. The total internal and total external resilience scores are averaged to obtain an overall resilience score. The possible range for all resilience scores (item scores, resilience subscale scores, total internal and external resilience scores and overall resilience score) is one to four. CHKS subscales have been found to be internally consistent and valid (internal resilience subscales: Cronbach’s α = 0.73 to 0.85; external resilience subscales: Cronbach’s *α* = 0.74 to 0.95) [19].

### Sample size

Results of past research [25,29] indicate that approximately 80% of students will participate in the survey. It is estimated that after accounting for a 25% attrition rate from baseline to follow-up, the cohort sample of interest will be composed of 1,360 Grade 7 students and 1,020 Grade 10 students in the control group, and 2,270 Grade 7 students and 1,700 Grade 10 students in the intervention group.

#### Primary outcome: risk of mental health problems

Using the above participant estimations, a cluster size of approximately 85 students per school is estimated. Based on a one-unit increase in total SDQ scores for control students (based on Australian norms indicating approximately a one-unit increase in score with age [55]), and a conservative estimate of a two-point reduction in total SDQ scores for intervention students (previous research indicates positive changes following intervention or treatment indicated by a reduction in self-report total SDQ score ranging from 4.93 [56] to 7.25 points [57]), it is estimated that approximately 11.6% more students at Grade 10 will score in the category of unlikely risk of mental health problems for the intervention group compared to the control group. The conversion from scores to percentages of students in the unlikely risk category was made using the SDQ frequency distribution for British 11 to 15 year olds, both sexes combined [58]. Based on an intra-cluster correlation coefficient of 0.037 [59,60], and the conservative assumption of independence of scores within a subject from Grades 7 to 10, the study will have 80% power to detect a difference of 11.6% between the two groups at Grade 10, at a 5% significance level.

### Statistical analysis

#### Analysis of demographic characteristics

To assess non-response bias, chi-squared analysis will be used to compare parental consent rates between intervention and control schools. Comparison of student demographic characteristics, for the intervention and control groups, will be completed at both baseline and follow-up using chi-squared analysis.

#### Analysis of primary outcome: risk of mental health problems

As recommended by the Australian Mental Health Outcomes and Classification Network [61], student total SDQs will be used to identify the proportion of consenting students at each school who are considered to be unlikely (total SDQ score 0 to 15), slightly (total SDQ score 16 to 19) and highly or significantly (total SDQ score 20 to 40) at risk of developing clinically significant mental health problems. Descriptive statistics will be used to report the proportion of students scoring in each risk category.

Intervention effectiveness will be assessed via the primary trial outcome using mixed models [62], under an intention-to-treat framework and using all available data. The primary outcome will be the proportion of students unlikely to be at risk of developing clinically significant mental health problems (total SDQ score <16) between Grade 10 students in intervention and control schools. Secondary outcomes will include analysis of the SDQ total and subscales both as scores and risks, the risks being examined as binary variables. The modelling approach will accommodate school clustering, and adjust for potential confounding effects (e.g. student or school characteristics). Sensitivity analyses will be carried out using pattern-mixture models. Subgroup analysis will be performed by gender. All data analysis will be conducted using the statistical program SAS [63].

## Discussion

In the authors’ knowledge, the present study is the first to investigate the effect of a resilience-based prevention-focused intervention in schools, utilising a randomised control study design, inclusive of a comprehensive measure of internal and external resilience factors, and incorporating intervention components into the school curriculum, environment and partnerships, for the proposed length of time, on reducing the risk of mental health problems in adolescents.

The mental health of young people is linked to many short- and long-term health outcomes. The findings of this research will add significantly to the understanding of the mental health of young people and has the potential to inform universal interventions to increase the positive mental health, resilience and life outcomes of adolescents. Implementing and evaluating school-based resilience interventions are of direct critical importance to students, teachers, mental health practitioners and policymakers [64].

## Trial status

The trial is ongoing and recruitment is not complete.

## Abbreviations

SEIFA: Socio-Economic Indexes for Areas; CHKS: California Healthy Kids Survey; NSW: New South Wales; SDQ: Strengths and Difficulties Questionnaire; total SDQ: total difficulties score.

## Competing interests

The authors declare that they have no competing interests.

## Authors’ contributions

JD drafted the manuscript and participated in the design and coordination of the study. MF, JB, EC and JW helped draft the manuscript; participated in a critical review of the manuscript content; and participated in the conception, design and coordination of the study. RKH participated in a critical review of the manuscript; and participated in the conception, design and coordination of the study. LW participated in a critical review of the manuscript; and participated in the conception and design of the study. All authors read and approved the final manuscript.
